# TheCellVision.org: A Database for Visualizing and Mining High-Content Cell Imaging Projects

**DOI:** 10.1534/g3.120.401570

**Published:** 2020-09-15

**Authors:** Myra Paz David Masinas, Mojca Mattiazzi Usaj, Matej Usaj, Charles Boone, Brenda J. Andrews

**Affiliations:** * The Donnelly Centre, University of Toronto, Ontario, M5S 3E1, Canada; † Department of Molecular Genetics, University of Toronto, Ontario, M5S 3E1, Canada

**Keywords:** high-content screening, quantitative image analysis, subcellular morphology, subcellular localization, protein abundance, penetrance, image analysis tools

## Abstract

Advances in genome engineering and high throughput imaging technologies have enabled genome-scale screens of single cells for a variety of phenotypes, including subcellular morphology and protein localization. We constructed TheCellVision.org, a freely available and web-accessible image visualization and data browsing tool that serves as a central repository for fluorescence microscopy images and associated quantitative data produced by high-content screening experiments. Currently, TheCellVision.org hosts ∼575,590 images and associated analysis results from two published high-content screening (HCS) projects focused on the budding yeast *Saccharomyces cerevisiae*. TheCellVision.org allows users to access, visualize and explore fluorescence microscopy images, and to search, compare, and extract data related to subcellular compartment morphology, protein abundance, and localization. Each dataset can be queried independently or as part of a search across multiple datasets using the advanced search option. The website also hosts computational tools associated with the available datasets, which can be applied to other projects and cell systems, a feature we demonstrate using published images of mammalian cells. Providing access to HCS data through websites such as TheCelllVision.org enables new discovery and independent re-analyses of imaging data.

High-content screening (HCS), which combines HTP microscopy with multi-parametric quantitative image analysis, has been used in recent years to explore different aspects of cell biology in diverse cell systems. HCS experiments produce massive image datasets, catalyzing an increasing demand for computational image analysis methods that enable extraction of quantitative information about a variety of cell features, including subcellular morphology, protein localization and abundance, and the dynamics of proteins or cellular compartments in response to genetic or environmental perturbations (Chong *et al.* 2015; de Groot *et al.* 2018; Heigwer *et al.* 2018; Mattiazzi Usaj *et al.* 2020; Mattiazzi Usaj *et al.* 2016; Styles *et al.* 2016; Yin *et al.* 2013). Several tools have been established in budding yeast that allow the exploration of gene and protein function using fluorescence microscopy. One key collection is the array of strains expressing GFP-fusion proteins (Huh *et al.* 2003) which enables proteome-scale exploration of protein abundance and localization (Breker *et al.* 2013; Chong *et al.* 2015; Denervaud *et al.* 2013; Kraus *et al.* 2017; Tkach *et al.* 2012). Collections of strains carrying deletions of non-essential yeast genes (Giaever *et al.* 2002) or temperature-sensitive alleles of essential genes (Costanzo *et al.* 2016; Li *et al.* 2011) can be modified using automated yeast genetics to express fluorescent reporters of compartments or biological processes of interest, or stained with fluorescent dyes, and then used in imaging screens to quantify the phenotypic changes associated with genetic perturbations (Eapen *et al.* 2017; Mattiazzi Usaj *et al.* 2015; Mattiazzi Usaj *et al.* 2020; Neumuller *et al.* 2013; Ohya *et al.* 2005; Sauerwald *et al.* 2015; Styles *et al.* 2016; Wolinski *et al.* 2009).

As noted above, high-content microscopy screens involve the acquisition of hundreds of thousands of images, from which single cell data are extracted and analyzed, producing several GBs to TBs of image data and associated numerical values. Recently, there have been important community efforts to develop standards and repositories for biological images and associated raw data (Orloff *et al.* 2012; Williams *et al.* 2017), which will accelerate re-analysis and higher-level mining of image datasets. However, the huge variety of biological images – including clinical, whole organism and single cell images – means that project- or organism-specific websites continue to serve a valuable complementary role. For example, several standalone websites allow users to search images of the yeast GFP-fusion collection (Breker *et al.* 2014; Dubreuil *et al.* 2019; Huh *et al.* 2003; Koh *et al.* 2015), with most accessible through the *Saccharomyces Genome Database* (SGD) (Cherry *et al.* 2012).

To examine subcellular phenotypes, we have performed several high-content screens using arrays of budding yeast mutants (Mattiazzi Usaj *et al.* 2020; Styles *et al.* 2016). Images and associated results from these and other studies that have systematically assessed (sub)cellular morphologies of single-gene mutant strains (Eapen *et al.* 2017; Mattiazzi Ušaj *et al.* 2015; Neumuller *et al.* 2013; Ohya *et al.* 2005; Sauerwald *et al.* 2015; Wolinski *et al.* 2009; Zhao *et al.* 2016) are often not incorporated into community databases, such as SGD, and thus they are less accessible for mining. Access to images and data from these types of HCS projects is needed to facilitate further analysis of the data, to allow for multi-project data mining to answer additional questions not addressed in primary publications, and to serve as benchmarking datasets for the development of novel image analysis methods. To address this need, we developed a centralized database and website, TheCellVision.org, that hosts both HCS projects employing the collection of GFP-fusion proteins and collections of single-gene mutant strains. In addition to the images and processed data obtained from HCS projects, we have made the analysis tools available on TheCellVision.org.

## Materials and Methods

### High-throughput imaging screens and analyses

At present, TheCellVision.org houses two published datasets:

- CYCLoPs (Collection of Yeast Cell and Localization Patterns). CYCLoPs consists of high-throughput imaging screens of yeast GFP-tagged strains, as well as the analysis of protein abundance and localization, which was obtained using support vector machines and convolutional neural networks, as described in Chong *et al.* and Kraus *et al.* (Chong *et al.* 2015; Kraus *et al.* 2017). The GFP collection was imaged in standard conditions, in the presence of 3 chemicals (HU – hydroxyurea; RAP – rapamycin, AF – alpha factor), and one mutant background (deletion of *RPD3* gene). The previous version of the CYCLoPs web-accessible database (Koh *et al.* 2015) has now been migrated to TheCellVision.org.
- Endocytic Compartment Morphology dataset. This dataset includes images from high-throughput screens of yeast single-gene mutants combined with single cell analysis of subcellular, endocytic compartment morphology, obtained using neural networks, as described in Mattiazzi Usaj *et al.* (Mattiazzi Usaj *et al.* 2020).

### Database development and schema

TheCellVision.org is a Django-based web application written in Python that allows access to multiple datasets obtained from various research projects. To store and manage large-scale data, TheCellVision.org uses a PostgreSQL object-relational database system on its backend. The web interface is developed using HyperText Markup Language (HTML5), Cascading Style Sheets (CSS3) and JavaScript. Open-source libraries such as Bootstrap and jQuery are utilized for easier implementation and to ensure an efficient web design across modern browsers. An nginx web server is used to process incoming client requests over Hypertext Transfer Protocol (HTTP) and serves responses through a Web Server Gateway Interface (uWSGI).

Currently, TheCellVision.org houses two research projects (see above), which together comprise ∼575,590 fluorescence microscopy images from ∼9,767 yeast strains and the associated quantitative data. The database schema is organized into three main clusters – one for each project and a core group which consists of tables that are shared by the projects (Figure S1). The database schema will continue to evolve as new experimental data are added to the database; the design is general and flexible, enabling the addition of new image datasets without database reconstruction.

### Analysis of the BBBC021 dataset

To test the image analysis tools developed for the Endocytic Compartment Morphology dataset (Mattiazzi Usaj *et al.* 2020) on an independent image dataset, we used the BBBC021v1 benchmarking set available from the Broad Bioimage Benchmark Collection (Caie *et al.* 2010; Ljosa *et al.* 2012). The dataset consists of fluorescence micrographs of MCF-7 breast cancer cells acquired on a high-content screening microscope (Caie *et al.* 2010). From the dataset, we collected subsets of images from a unique compound-concentration combination for each of 13 phenotypic classes (Caie *et al.* 2010).

CellProfiler (Carpenter *et al.* 2006) was used for object segmentation and extraction of cellular features such as area, shape, intensity, texture, granularity and radial distribution. The segmentation pipeline is available at https://data.broadinstitute.org/bbbc/BBBC021/ (section ‘CellProfiler pipelines’). To segment the cells, primary objects (nuclei) were first identified from the DNA channel, then the F-actin channel was used to identify the secondary objects (cells). Lastly, to determine cytoplasmic regions, each identified nucleus was subtracted from its corresponding cell object. Cells touching the border of the image were discarded. In total, we extracted quantitative information for 22,529 cells from 160 image sets (∼1.2% of the dataset). The single cell classification tool used for analysis of the Endocytic Compartment Morphology dataset (2-hidden-layer fully connected neural network) was then applied to the extracted data (Mattiazzi Usaj *et al.* 2020).

### Data availability

Bulk download of images from the CYCLoPs dataset will be provided upon request. Raw and processed images associated with the Endocytic Compartment Morphology dataset were deposited to the Image Data Resource (https://idr.openmicroscopy.org) under accession number idr0078. Datasets can be downloaded from each project’s ‘Supplemental Files’ tab. This includes protein localization and abundance, penetrance and phenotype data. Details on the CYCLoPs datasets and the Endocytic Compartment Morphology dataset are described elsewhere (Chong *et al.* 2015; Kraus *et al.* 2017; Mattiazzi Usaj *et al.* 2020).

Code for the 2 hidden-layer fully connected neural network for single cell classification, and other developed tools is available at https://thecellvision.org/tools.

All supplemental material is available at figshare. Figure S1 illustrates the database schema for each of the clusters: A) core, B) Endocytic Compartment Morphology project, C) CYCLoPs project. Table S1 provides the number of cells for each class of the BBBC021 dataset included in the analysis, classification accuracy for each class, and the effect of training set size on classification accuracy. Supplemental material available at figshare: https://doi.org/10.25387/g3.12950441.

## Results and Discussion

### Image collections available on TheCellVision.org

**Figure 1:**
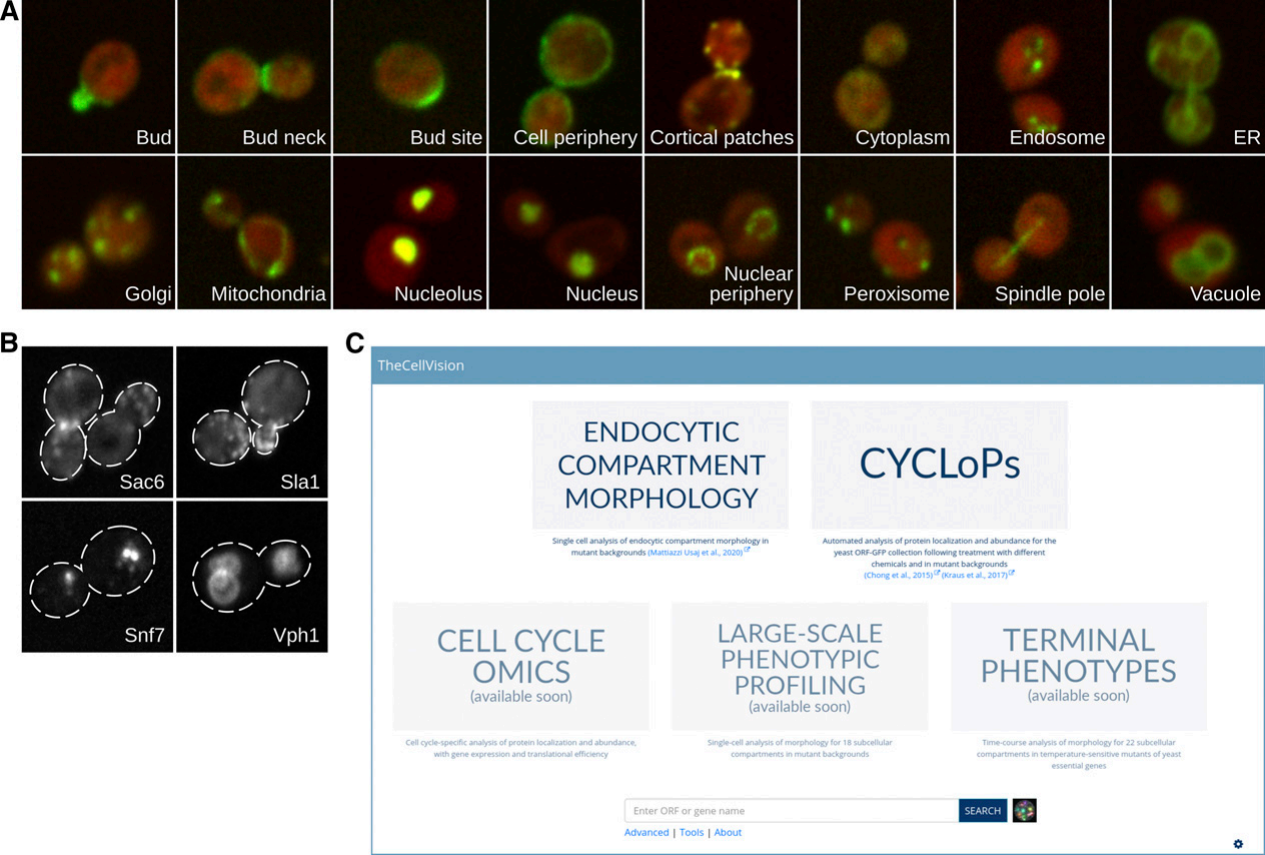
A) Example micrographs of the 16 localization classes of the Collection of Yeast Cell and Localization Patterns dataset (CYCLoPs). B) Example micrographs of the 4 endocytic markers of the Endocytic Compartment Morphology dataset. C) TheCellVision.org landing page.

The first release of TheCellVision.org houses two large-scale datasets: the Collection of Yeast Cell and Localization Patterns dataset (CYCLoPs) (Chong *et al.* 2015; Kraus *et al.* 2017), and the Endocytic Compartment Morphology dataset (Mattiazzi Usaj *et al.* 2020). The CYCLoPs dataset comprises 385,984 images: 330,688 images from 18 screens described in Chong *et al.* (Chong *et al.* 2015), and 55,296 images from an alpha-factor time-course screen described in Kraus *et al.* (Kraus *et al.* 2017). These are 2-channel images: the green (GFP) channel represents a GFP-fused protein from the ORF-GFP collection (Huh *et al.* 2003), while the red (RFP) channel identifies the cytosol and was used for cell segmentation purposes. In total the CYCLoPs dataset covers 4,139 proteins from *S. cerevisiae*. These proteins have been classified into 16 localization classes (Figure 1A) (Chong *et al.* 2015).

The Endocytic Compartment Morphology dataset comprises 189,606 images, which are single-channel maximum z-projections depicting the subcellular morphology of 4 endocytic markers (Figure 1B) in 5,628 yeast mutant strains (corresponding to 5,267 *S. cerevisiae* ORFs). The screened mutant strains include non-essential gene deletion strains, and strains with temperature-sensitive alleles of essential genes. All temperature-sensitive strains were screened at two temperatures (26° and 37°). The identification of significant morphology mutants only included temperature-sensitive strains screened at the non-permissive temperature (37°). However, all screens done at the permissive temperature (26°) are included in TheCellVision.org together with micrographs, and results from penetrance and phenotype analyses.

### TheCellVision.org functionalities

Our aim was to design a database that provides the user with a simple yet informative interface for accessing data from our image-based yeast screens. The landing page contains icons for each of the main research projects that lead to their respective home page (Figure 1C). From the landing page, the user can also access the image analysis tools (‘Tools’), and an advanced search option (‘Advanced’). In addition, a search box allows the user to search for genes of interest across all open projects. Clicking on TheCellMap.org icon on the right side of the search bar will open a separate window displaying the genetic interaction results for the queried genes on our sister site, TheCellMap.org, which hosts data associated with the global yeast synthetic genetic interaction mapping project (Costanzo *et al.* 2016; Usaj *et al.* 2017).

#### Search box and general search results:

**Figure 2:**
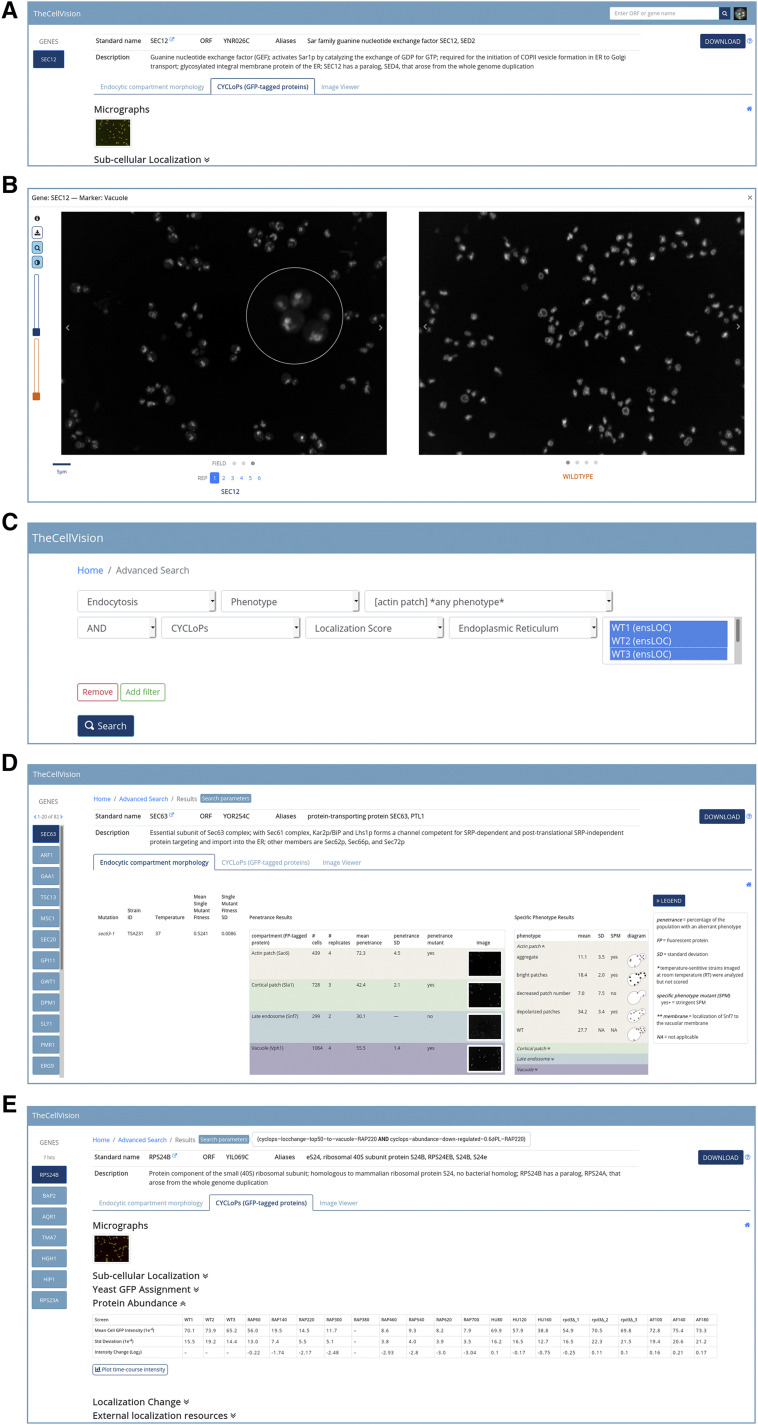
TheCellVision.org functionalities. A) Example results page of a simple search. B) Clicking on a thumbnail opens up an image modal with the enlarged image. Among the features available in the modal are the magnifying glass and brightness adjustment. C) The advanced search allows the user to search for more complex combinations of terms across different projects and/or screens using logical AND, OR, NOT operations. D) Results of the example advanced search from C). E) Example of advanced search results combining Localization change and Protein abundance data from the CYCLoPs dataset.

Typing into the search box on the landing page will prompt the user with a list of available matching gene names. Alternatively, the user also has the option to directly paste in a list of genes. Clicking on the search icon triggers the request to retrieve available data associated with the input genes across all projects. The search results page displays the input list of genes on the left pane. On top of the results page is a brief summary of the selected gene with a link to the gene’s summary page on SGD. For easier navigation between projects, the results page has a tab-separated interface. Searching for a gene that has not been screened will return a “No results found” message in the main results window. (Figure 2A).

The Endocytic Compartment Morphology tab provides basic information on the imaged strain with the number of cells and replicates included in the analysis, penetrance data for the 4 screened markers, fraction of cells with each phenotype for the 17 mutant phenotypes (collapsible table), and all available microscopy images, including examples of wild-type strain micrographs. A brief legend can be accessed by clicking on the collapsible ‘Information’ icon. On the CYCLoPs tab, the displayed data include information on protein subcellular localization and abundance, as well as localization changes in the tested conditions (described in Chong *et al.* (Chong *et al.* 2015)), and micrographs from all CYCLoPs screens for the searched strain. Links to external protein localization databases are included at the bottom of the page. CYCLoPs data can also be accessed through SGD’s Localization Resources. Finally, a download button is available in the top right corner which allows the user to download a spreadsheet containing the data provided on the results page for all queried genes. An information icon beside the download button acts as a link to a separate page listing the column information for the downloaded file.

#### Image modals:

The image thumbnails displayed on the results page are in PNG format, and are generated instantaneously for each searched gene from original TIF images stored in the database. Clicking on a thumbnail opens up a modal with the enlarged image (Figure 2B). The modal’s header provides general information on the strain and screen being displayed (*e.g.*, *SEC12*, vacuole in the example shown in Figure 2A). A magnifying glass icon is available on the left side of the modal. Once the icon is clicked and the user hovers the pointer over the image, the pointer transforms into a magnifying glass which allows the user to zoom into cells of interest (Figure 2B). By default, the images have their intensity values scaled to the maximum pixel value in the image. Clicking on the ‘Adjust brightness’ icon opens two sliders for adjusting the image brightness: the brightness settings on each slider will be propagated to all images of the selected replicate of the searched mutant stain and all displayed control wild-type strain images, respectively. Navigation buttons at the bottom of the displayed image can be used to browse through individual images in all available replicates. For the Endocytic Compartment Morphology project, the mutant (left) and wild-type images from the same screen (right) are displayed side by side for phenotype comparison (Figure 2B). The displayed wild-type images are randomly selected from the same imaging plate as the mutant. To download the set of images for the currently selected strain and marker as a single .zip file, the user can click the ‘Download images’ icon. The downloaded images are all 16-bit uncompressed grayscale TIFs.

For CYCLoPs, toggle buttons are available for viewing individual fluorescence channels. By default, an overlay of both channels is shown. In addition, the user can enable the dual view (eye icon) that allows side-by-side comparison of images from different CYCLoPs screens.

#### Image viewer:

For a more comparative visualization of images, an ‘Image Viewer’ tab is included on the general results page. For a selected gene, the image viewer displays a gallery of all available micrographs across all projects, including mutants of the gene with various endocytic compartments tagged with fluorescent proteins and GFP-tagged versions of the encoded protein. Alternatively, a gallery of all available micrographs across all searched genes for a selected project may be viewed (‘All genes’ option). The magnifying glass and brightness adjustment icons can be found on the top right side.

#### Advanced search:

From the main landing page, clicking the ‘Advanced’ link takes the user to the advanced search page. This page allows the user to search for more complex combinations of terms across different projects and/or screens using logical AND, OR, NOT operations (Figure 2C). The user can select from a list of projects, and predefined project-specific data types, markers, screens, phenotypes, or localizations. For example, a user might want to ask whether any endoplasmic reticulum proteins are important for proper actin patch morphology. In this case, a search for genes that when mutated cause a defect in actin patch morphology, and whose products localize to the endoplasmic reticulum returns 82 genes (Figure 2D) indicating that many ER proteins play an important role in actin patch morphology. For search results involving more than 20 genes, a pagination bar appears on top of the gene list that allows the user to navigate between pages.

The advanced search can also combine different data types from the same project. For example, using the CYCLoPs dataset the user can look for proteins that go to the vacuole for degradation after rapamycin treatment (example input parameters for this search are shown in Figure 2E). The search returns a list of 7 proteins that are involved in translation or nutrient transport (Figure 2E) – inhibition of these processes is consistent with known effects of TOR inhibition with rapamycin (Loewith and Hall 2011).

#### Image analysis tools:

The field of bioimage analysis has seen rapid growth in recent years. There are several available software tools that perform different image processing and analysis steps (Smith *et al.* 2018). All the tools and code developed as part of the projects hosted by TheCellVision.org are available in the ‘Tools’ tab. The link next to each tool will take the user to its respective GitHub page. These tools can be adapted for the analysis of other fluorescent markers and subcellular compartments, and can also be used, as we show below, to analyze images in a species-independent manner.

**Figure 3:**
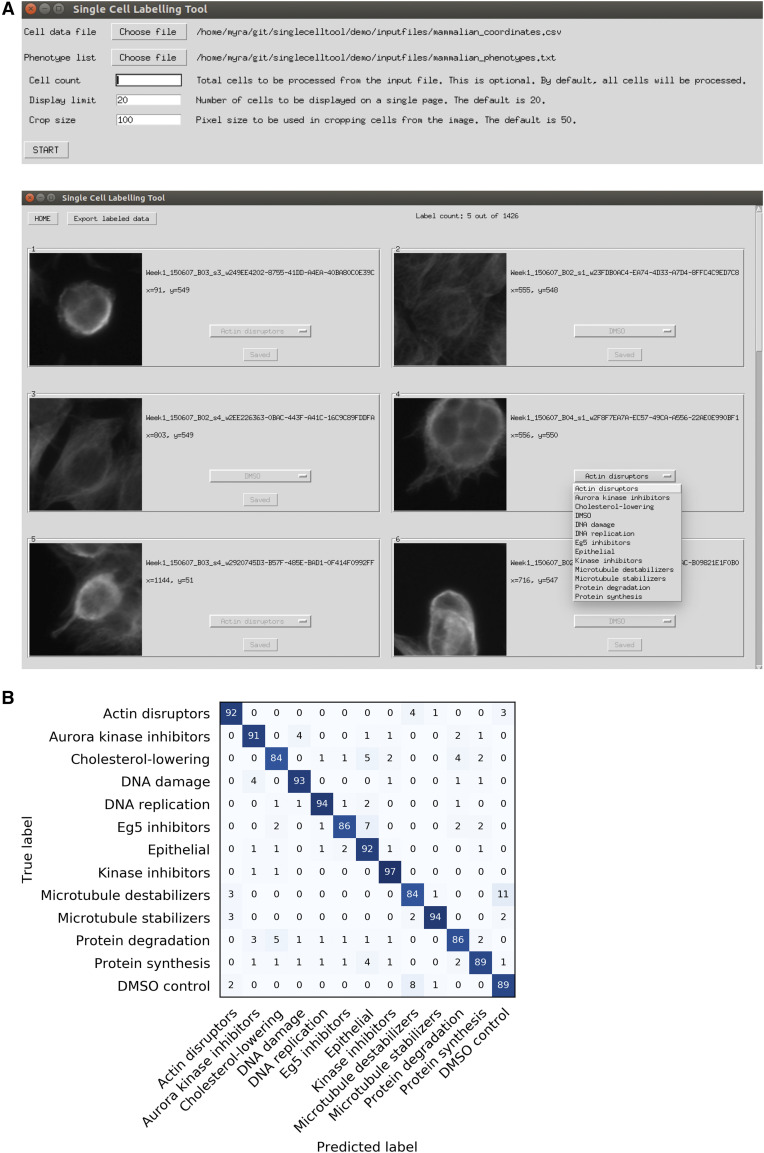
Image analysis tools available through TheCellVision.org. A) The single cell labeling tool offers a simple way to manually annotate single cells when building a classification training set. Top: Input GUI. Bottom: Labeling tool GUI. B) Confusion matrix illustrating the classification accuracy of the 2-hidden-layer fully connected neural network for the 13 classes of the BBBC021 dataset.

One of the tools, the single cell labeling tool, offers a simple way to manually annotate single cells when building a classification training set (Figure 3A). This graphical user interface (GUI) application allows the user to view and label single cell images on a grid layout. The required input is a simple spreadsheet containing single cell data, such as image path location and cell coordinates, and a list of possible labels. The user can annotate single cells with one of the labels and export the classification training set. This classification training set can be used in combination with the 2-hidden-layer fully connected neural network (ODNN in ‘Tools’ in TheCellVision.org) developed in Mattiazzi Usaj *et al.* (Mattiazzi Usaj *et al.* 2020) to classify phenotypes.

Because the inputs are quantitative single cell descriptors, we predicted that the 2-hidden-layer fully connected neural network classifier could be used for the analysis of both yeast and other cells. To test the usability and performance of the classifier on human cell images, we took advantage of the BBBC021 benchmarking set available from the Broad Bioimage Benchmark Collection (Caie *et al.* 2010; Ljosa *et al.* 2012). The dataset consists of fluorescence micrographs of MCF-7 human breast cancer cells acquired on a high-content screening microscope. We first tested the performance of the classifier on a set of 22,529 cells extracted from only ∼1.2% of images of the BBBC021 dataset and achieved ∼85% classification accuracy without any hyper-parameter optimization (Table S1). We next asked if we could get better classification accuracy by removing mislabeled cells from the training set. In the dataset, all cells from a given compound-concentration combination are assigned the same mode of action (MOA) label, but all cells do not actually display the same phenotype. We excluded from the dataset 1474 objects (∼6.5%) that were classified to a different MOA class in more than 50% of a total of 150 predictions from 3 independent runs. The classification accuracy for the cleaned training set was ∼90% (Figure 3B). The final number of cells for each class in the cleaned dataset is available in Table S1. We also tested the effect of training set size on classification accuracy. Three independent runs were performed for training sets comprised of 50 to 1000 cells per class (Table S1). Similarly to the Endocytic Compartment Morphology dataset (Mattiazzi Usaj *et al.* 2020), we observed the largest differences in classification accuracy between smaller training sets; training sets with sizes of 500 cells or more showed modest increases in classification accuracy (4% higher accuracy with training sets of 1000 cells compared to that of 500 cells).

In summary, we show that our 2-hidden-layer fully connected neural network classifier accurately classifies at least two disparate cell types, yeast and human cells (Figure 3B and Mattiazzi Usaj *et al.* (Mattiazzi Usaj *et al.* 2020)). We therefore predict that our single cell labeling tool and classifier will be useful for analyzing image data from different cell types.

#### Individual project pages:

Each project has a different customized interface for browsing the analysis results and images for the searched genes. The results of individual projects can be accessed either through the general search (as described above) or through the project’s home page. On each project’s home page, the user can also access project-specific information and help pages (‘About’, ‘Help’), the single-project advanced search option (‘Advanced search’), and any supplemental files published with the primary paper as well as additional unpublished data (‘Supplemental files’). For example, the additional files available for the Endocytic Compartment Morphology project include the classification training sets (quantitative features for all single cells used for training), phenotype profiles of the identified morphology mutants and pairwise phenotype profile similarities (Mattiazzi Usaj *et al.* 2020).

## Conclusions and future directions

Each high-throughput imaging screen produces thousands of images, with gigabytes of data. TheCellVision.org provides single-platform interactive browsing of images and data generated with different high-throughput imaging screens and projects and is the first database that allows a multi-parametric search across results from different HCS projects. The current implementation houses two large-scale projects performed in budding yeast, comprising ∼575,590 images, as well as the associated image analysis tools. The developed tools can be used in the analysis of different cell types (see section *Image analysis tools*), and we anticipate TheCellVision.org will provide a valuable resource to members of the community.

We continue to generate new datasets that answer diverse biological questions, and new data, including non-yeast data, will be deposited in TheCellVision.org as it is ready for release. Upcoming datasets include ‘Cell Cycle Omics’ - cell cycle-specific analysis of protein localization and abundance, with gene expression and translational efficiency data, ‘Large-scale Phenotypic Profiling’ - single-cell analysis of morphology for 18 subcellular compartments in mutant backgrounds, and ‘Terminal Phenotypes’ - time-course analysis of morphology for 22 subcellular compartments in temperature-sensitive mutants of yeast essential genes. In addition, we are further developing the advanced search function that allows the user to simultaneously search across different projects, and we are expanding the set of image analysis tools. In the future, we plan on adding a ‘multi-omics’ search option that would allow the user to search genes of interest across TheCellVision.org and its sister-site, TheCellMap.org, that houses synthetic genetic interaction data (Usaj *et al.* 2017). The database will be a valuable tool for biologists interested in exploring different aspects of eukaryotic cell biology, and those looking for simple ready-to-use tools to analyze their image dataset, as well as for researchers developing image analysis tools in need of new benchmarking datasets.
